# COVID-19 and Ventricular Shunt Revisions

**DOI:** 10.7759/cureus.27059

**Published:** 2022-07-20

**Authors:** David R Hallan, Elias Rizk

**Affiliations:** 1 Neurosurgery, Penn State Health Milton S. Hershey Medical Center, Hershey, USA

**Keywords:** covid-19, mortality rate, shunt revision, outcomes, ventriculoperitoneal shunt, shunt, covid, hydrocephalus, neurosurgery

## Abstract

Introduction: COVID-19 patients frequently experience headaches, malaise, and fatigue. For patients with shunted hydrocephalus, these signs and symptoms can often be indicative of shunt failure. Thus, it can be challenging to determine if shunt failure has occurred in this patient population. Therefore, we explored the question of how a diagnosis of COVID-19 in shunted hydrocephalus patients influences the rate of shunt revision.

Methods: We used a deidentified database network (TriNetX) to gather information on patients with shunted hydrocephalus and COVID-19 versus no COVID-19 from January 20, 2020, through September 26, 2021. Our primary outcome of interest was shunt revision at 90 days, with secondary outcomes of mortality, hospitalization, ICU admission, mechanical ventilation, tracheostomy, PEG tube placement, fall, seizure, acute kidney injury (AKI), venous thromboembolism (VTE), ischemic stroke (I.S.), myocardial infarction (MI), and sepsis. Cohorts were propensity score-matched for common comorbidities and demographics.

Results: After propensity score matching, 10,600 patients with shunted hydrocephalus and COVID-19 (cohort 1) and 10,600 patients with shunted hydrocephalus and no COVID-19 (cohort 2) were identified. Average age was 38.5 years. Eight hundred and thirty-four patients (7.869%) in cohort 1 and 180 (1.698%) patients in cohort 2 underwent shunt revision (p=<0.0001, OR 4.978, 95% CI 4.198, 5.821). Mortality was 4.642% vs. 2.113% (p<0.0001, OR 2.255, 95% CI 1.921, 2.647). Hospitalization rates were 27.72% vs. 10.303% (p<0.0001), and ICU admission rates 11.567% vs. 3.463% (p<0.0001). Ventilator dependence was 3.529% vs. 0.953% (p<0.0001), tracheostomy 1.142% vs. 0.236% (p<0.0001), PEG tube insertion 2.472% vs. 0.585% (p<0.0001), falls 2.321% vs. 1.076% (p<0.0001), seizure 11.369% vs. 5.953% (p<0.0001), AKI 4.416% vs. 1.717% (p<0.0001), VTE 3.538% vs. 1.293% (p<0.0001), sepsis 3.887% vs. 1.179% (p<0.0001), IS 0.585% vs. 0.16% (p<0.0001), and MI 1.34% vs. 0.519% (p<0.0001).

Conclusion: COVID-19 infection is associated with an almost five-fold increase in shunt revisions.

## Introduction

COVID-19 patients frequently experience headaches, malaise, and fatigue [1-5]. For patients with shunted hydrocephalus, these signs and symptoms can often be indicative of shunt failure. Thus, it can be difficult to determine if shunt failure has occurred in this patient population. We explored the question of how a diagnosis of COVID-19 in shunted hydrocephalus patients influences the rate of shunt revision using a multicenter research network with matched controls.

## Materials and methods

This was a retrospective comparative case-control study. We used a de-identified database network (TriNetX) to retrospectively query via ICD-10 and current procedural terminology codes to evaluate all patients with a diagnosis of COVID-19 and a shunt (cohort 1) versus no COVID-19 and a shunt (cohort 2). Data came from 62 health care organizations (HCOs). Data includes demographics, diagnoses, medications, laboratory values, genomics, and procedures. The identity of the HCOs and patients is not disclosed to comply with ethical guidelines against data re-identification. Because of the database's federated nature, an IRB waiver was granted. The data is updated daily. Our use of this database and its validity were informed by previous literature, and exact details of the network have been previously described [6-9]. Diagnosis of COVID-19 was based on ICD-10 codes (U07.1, U07.2, J12.81, B34.2, B97.21, B97.29) and/or SARS-CoV-2 polymerase chain reaction (PCR) positivity. Data spanned January 20, 2020 - September 21, 2021. The index date was set at the date of COVID-19 diagnosis with a shunt (cohort 1) versus a shunt (cohort 2). Our primary outcome of interest was shunt revision at 90 days, with secondary outcomes of mortality, hospitalization, ICU admission, mechanical ventilation, tracheostomy, percutaneous endoscopic gastrostomy (PEG) tube placement, fall, seizure, acute kidney injury (AKI), venous thromboembolism (VTE), ischemic stroke (I.S.), myocardial infarction (MI), and sepsis.

The medical information included age at index date, as well as sex, race, and comorbidities of hypertension, acute kidney injury, diabetes, ischemic heart disease, heart failure, atrial fibrillation, disorders of lipoprotein metabolism and other lipidemias, obesity, history of nicotine dependence, chronic respiratory disease, cirrhosis, alcohol abuse or dependence, and peripheral vascular disease, recorded up to the date of the index date. Analysis was performed using unmatched and propensity score-matched cohorts, with the greedy-nearest neighbor algorithm with a caliper of 0.1 pooled standard deviations. Hazard ratios were calculated using R's survival package v3.2-3 and validated, comparing the output to SAS version 9.4. Chi-square analysis was performed on categorical variables. Significance was defined as a p-value less than 0.05.

## Results

A total of 12,235 patients with shunted hydrocephalus and who were COVID-19 positive were identified, versus 14,944 with a shunt and no COVID-19. After propensity score matching, 10,599 patients were identified in each cohort. After matching, age at index was 38.5+-25.9 and 38.4+-25.6 for cohorts 1 and 2, respectively. 48.901% of cohort 1 were male, and 48.903% were in cohort 2. 68.19% vs. 66.147% of patients were white, 19.224% vs. 17.131% were black or African American, and 1.332% vs. 1.470% were Asian. Baseline demographics and characteristics are shown in Table 1. The table also includes ICD-10 codes.

**Table 1:** Baseline demographics and characteristics after propensity score matching Cohort 1: COVID-19 and a ventricular shunt Cohort 2: Ventricular shunt without COVID-19

		Before Matching			After Matching		
Code	Diagnosis	Cohort 1, n (%)	Cohort 2, n (%)	Std diff.	Cohort 1, n (%)	Cohort 2, n (%)	Std diff.
AI	Age at Index	39.39 (100)	38.23 (100.00)	-	38.54 (100.00)	38.46 (100.00)	-
2106-3	White	8328 (68.16)	9900 (66.17)	0.040	7223 (68.14)	7256 (68.45)	0.0066
F	Female	6241 (51.08)	7642 (51.08)	0.00	5406 (51.00)	5374 (50.69)	0.0060
M	Male	5974 (48.89)	7317 (48.91)	0.0002	5191 (48.97)	5224 (49.28)	0.0062
2054-5	Black or African American	2352 (19.25)	2560 (17.111)	0.056	1937 (18.27)	1933 (18.24)	0.00098
2131-1	Unknown Race	1323 (10.83)	2205 (14.74)	0.12	1244 (11.74)	1223 (11.54)	0.0062
2028-9	Asian	163 (1.33)	221 (1.48)	0.012	147 (1.39)	139 (1.31)	0.0065
I10-I16	Hypertensive diseases	5005 (40.96)	4247 (28.39)	0.27	3790 (35.76)	3869 (36.50)	0.016
R53	Malaise and fatigue	3927 (32.14)	3092 (20.67)	0.26	2846 (26.85)	2903 (27.39)	0.012
E78	Disorders of lipoprotein metabolism and other lipidemias	3094 (25.32)	2631 (17.59)	0.19	2292 (21.62)	2343 (22.10)	0.012
R63	Symptoms and signs concerning food and fluid intake	3024 (24.75)	2225 (14.87)	0.25	2182 (20.59)	2176 (20.53)	0.0014
J40-J47	Chronic lower respiratory diseases	2723 (22.29)	2261 (15.11)	0.18	1996 (18.83)	2045 (19.29)	0.012
R13	Aphagia and dysphagia	2850 (23.33)	1947 (13.01)	0.27	1952 (18.42)	1898 (17.91)	0.013
R40	Somnolence, stupor and coma	2271 (18.59)	1524 (10.19)	0.24	1507 (14.22)	1471 (13.88)	0.0098
E08-E13	Diabetes mellitus	1973 (16.15)	1533 (10.25)	0.18	1414 (13.34)	1411 (13.31)	0.00083
N17-N19	Acute kidney failure and chronic kidney disease	2122 (17.37)	1338 (8.94)	0.25	1344 (12.68)	1311 (12.37)	0.0094
Z87.891	Personal history of nicotine dependence	1672 (13.69)	1407 (9.40)	0.13	1240 (11.69)	1244 (11.74)	0.0012
F17	Nicotine dependence	1470 (12.03)	1286 (8.59)	0.11	1118 (10.55)	1097 (10.35)	0.0065
I20-I25	Ischemic heart diseases	1498 (12.26)	1016 (6.79)	0.19	1006 (9.49)	975 (9.19)	0.010
I50	Heart failure	941 (7.70)	572 (3.82)	0.17	568 (5.36)	556 (5.25)	0.0051
I48	Atrial fibrillation and flutter	760 (6.22)	556 (3.72)	0.12	526 (4.96)	504 (4.76)	0.0097
I73	Other peripheral vascular diseases	502 (4.11)	338 (2.26)	0.11	321 (3.028)	317 (2.99)	0.0022
F10.1	Alcohol abuse	344 (2.82)	221 (1.48)	0.092	216 (2.038)	217 (2.05)	0.00067
F10.2	Alcohol dependence	218 (1.78)	150 (1.00)	0.067	140 (1.32)	133 (1.26)	0.0059
K74	Fibrosis and cirrhosis of liver	158 (1.29)	85 (0.57)	0.076	90 (0.85)	85 (0.80)	0.0052

Eight hundred and thirty-four patients (7.869%) in cohort 1 and 180 (1.698%) patients in cohort 2 underwent shunt revision (p=<0.0001, OR 4.978, 95% CI 4.198, 5.821). Mortality was 4.642% vs. 2.113% (p<0.0001, OR 2.255, 95% CI 1.921, 2.647). Hospitalization rates were 27.72% vs. 10.303% (p<0.0001), and ICU admission rates 11.567% vs. 3.463% (p<0.0001). Ventilator dependence was 3.529% vs. 0.953% (p<0.0001), tracheostomy 1.142% vs. 0.236% (p<0.0001), PEG tube insertion 2.472% vs. 0.585% (p<0.0001), falls 2.321% vs. 1.076% (p<0.0001), seizure 11.369% vs. 5.953% (p<0.0001), AKI 4.416% vs. 1.717% (p<0.0001), VTE 3.538% vs. 1.293% (p<0.0001), sepsis 3.887% vs. 1.179% (p<0.0001), IS 0.585% vs. 0.16% (p<0.0001), and MI 1.34% vs. 0.519% (p<0.0001). This is summarized in Table 2.

**Table 2:** Outcomes after propensity score matching Cohort 1: COVID-19 and a ventricular shunt Cohort 2: Ventricular shunt without COVID-19

Outcome	Cohort 1, n (%)	Cohort 2, n (%)	Odds ratio (95% CI)	P-value
Mortality	492 (4.64)	224 (2.11)	2.26 (1.92,2.65)	<0.0001
Shunt revision	834 (7.87)	180 (1.69)	4.94 (4.19,5.82)	<0.0001
Ventilator dependence	374 (3.53)	101 (0.95)	3.80 (3.047,4.74)	<0.0001
Tracheostomy	121 (1.14)	25 (0.24)	4.88 (3.17,7.52)	<0.0001
PEG	262 (2.47)	62 (0.59)	4.31 (3.26,5.69)	<0.0001
Falls	246 (2.32)	114 (1.08)	2.19 (1.75,2.73)	<0.0001
Seizures	1205 (11.37)	631 (5.95)	2.02 (1.83,2.22)	<0.0001
Hospitalization	2938 (27.72)	1092 (10.30)	3.34 (3.09,3.60)	<0.0001
Intensive care unit	1226 (11.57)	367 (3.46)	3.65 (3.23,4.11)	<0.0001
Acute kidney injury	468 (4.42)	182 (1.72)	2.64 (2.22,3.15)	<0.0001
Venous thromboembolism	375 (3.54)	137 (1.29)	2.80 (2.29,3.41)	<0.0001
Sepsis	412 (3.887)	125 (1.18)	3.39 (2.77,4.15)	<0.0001
Ischemic stroke	62 (0.59)	17 (0.16)	3.66 (2.14,6.27)	<0.0001
Myocardial infarction	142 (1.34)	55 (0.52)	2.60 (1.91,3.56)	<0.0001

**Figure 1:**
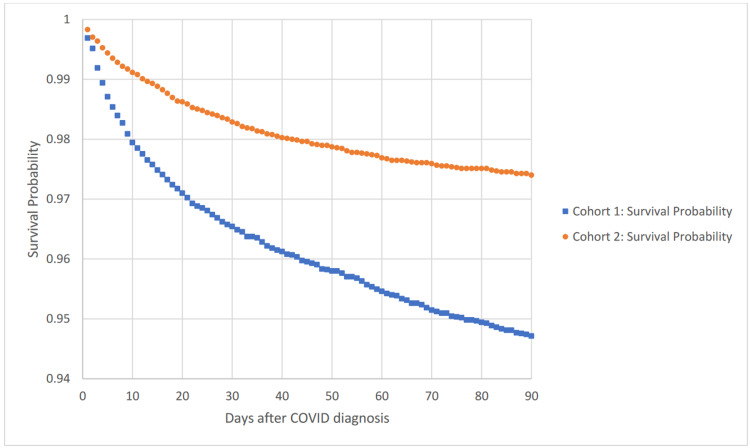
Kaplan-Meier survival analysis for outcome: deceased Cohort 1: COVID-19 and a ventricular shunt Cohort 2: Ventricular shunt without COVID-19

Figure 1 shows a Kaplan-Meier survival curve for outcome deceased to 90 days comparing cohorts 1 and 2. The hazard ratio was 2.035, with 95% CI 1.737, 2.383, p<0.0001.

## Discussion

Our results demonstrate a significant increase in shunt revisions associated with COVID-19 infection. As COVID-19 infection has been reported to cause neurological symptoms such as headache, weakness, altered mental status, seizure, stroke, drowsiness, malaise, fatigue, hypotonia, and peripheral neuropathy, and these symptoms can often be found in shunt failure, it begs the question on if COVID-19 is causing shunts to malfunction, or the infection is masquerading as a shunt malfunction [1,4,10]. Furthermore, rates of avoidable shunt revisions are high [11,12]. It is, therefore, important that clinicians become well-versed in the neurologic manifestations and complications of COVID-19 [13].

Silva et al. in 2020 reported 56 patients who had headaches and COVID-19 and subsequently underwent lumbar puncture (L.P.) for cerebrospinal fluid (CSF) analysis. Eleven of the 56 patients had an opening pressure of greater than 200 mmH2O, six of which had pressures greater than 250 mmH2O. Two patients had papilledema. All patients had normal CSF analysis. They concluded that COVID-associated coagulopathy might be an explanation for the increased intracranial pressure (ICP) [4].

Baccarella et al., in 2021, reported a case series of two patients with multi-inflammatory syndrome associated with COVID-19 infection who had evidence of increased intracranial pressure. The first patient presented with a headache, a right abducens palsy, and no papilledema. An L.P. was performed and revealed an opening pressure of 34 cm H2O and CSF without abnormality. The patient's headache improved after the L.P. The second patient also presented with a headache and was found to have bilateral papilledema and right abducens palsy. An L.P. was performed one week after the patient presented and revealed an opening pressure of 14 cm H2O and CSF without abnormality. The authors concluded that the multi-inflammatory syndrome associated with COVID-19 might cause increased ICPs, as has been noted in other systemic inflammatory disorders such as Lupus, Sjogren's, and Kawasaki disease [5].

Eden et al. in 2020 published a case series of CSF biomarkers in patients with neurological symptoms who also were COVID-19 positive. They found that CSF neopterin, a marker of inflammation, was elevated in all patients. They likewise noted that CSF beta-2-microglobulin was similarly elevated in all CSF samples. CSF neurofilament light chain protein, a marker of axonal injury, was elevated in one-third of patients, and SARS-CoV-2 RNA was also found in the CSF of one-third of patients. The white blood cell count, albumin ratios, and immunoglobulin G were all normal. This suggested that COVID-19 likely causes inflammation and possible axonal disruption of the central nervous system [2].

Lewis et al., in 2021, published a literature review of CSF in COVID-19-positive patients. Overall, the literature examined reported 430 patients with neurologic symptoms that prompted CSF testing. The authors found that SARS-CoV-2 in CSF is rare, at 6%. In addition, they found that 7% of patients had elevated WBC count in the CSF, and 40% of patients had elevated protein, indicative of inflammation or axonal injury. Thus, the authors conclude that most neurological complications of COVID-19 were unlikely related to direct viral neuroinvasion but instead could be due to toxic-metabolic changes, hypoxic-ischemic injury, and/or inflammatory response [3].

One recent paper by Laxpati et al. examined the number of shunt surgeries performed during the COVID-19 pandemic. This study was done at a single institution, looking at the number of elective and emergent shunt revisions from 2015 to 2020. They found that during 28 days in March of 2020, only 32 shunt surgeries were performed. As compared to numbers before the onset of the COVID-19 pandemic, this was a statistically significant decrease in the number of surgeries performed. The authors concluded that there was an environmental factor related to the pandemic that might be altering the presentation rate of shunt malfunctions [14]. A 2022 study by Lee et al. likewise showed a decreased total number of neurosurgery emergency patients from January 2020 to September 2020 [15]. 

Our analysis was not without limitations. The major limitation of this study was that it was retrospective. Furthermore, due to the nature of the database, we were unable to collect patient-level data on specific outcomes. We were unable to report on radiology information. We do not have information on the type of diagnostic test used for confirmation of disease. We do not have information on the type of shunt malfunction (proximal, distal, valve, or no shunt malfunction) found intraoperatively. In addition, some misidentification is inevitable in database studies.

## Conclusions

COVID-19 is associated with an almost five-fold increase in shunt revisions. Likewise, patients with shunts who are COVID positive had higher mortality rates, hospitalization, ICU admission, ventilator dependence, tracheostomy, PEG tube insertion, falls, seizures, AKI, VTE, sepsis, I.S., and MI. This increase in shunt revisions could be due to associated increases in intracranial pressures from COVID-19 infection related to direct viral neuroinvasion, toxic-metabolic changes, multiinflammatory syndrome, coagulopathy, or hypoxic-ischemic injury.
